# Dietary Patterns and Metabolic Syndrome in Adult Subjects: A Systematic Review and Meta-Analysis

**DOI:** 10.3390/nu11092056

**Published:** 2019-09-02

**Authors:** Roberto Fabiani, Giulia Naldini, Manuela Chiavarini

**Affiliations:** 1 Department of Chemistry, Biology and Biotechnology, University of Perugia, 06123 Perugia, Italy; 2 School of Specialization in Hygiene and Preventive Medicine, Department of Experimental Medicine, University of Perugia, 06123 Perugia, Italy; 3 Department of Experimental Medicine, Section of Public Health, University of Perugia, 06123 Perugia, Italy

**Keywords:** dietary pattern, metabolic syndrome, systematic review, meta-analysis

## Abstract

Metabolic Syndrome (MetS) constitutes a relevant public health burden. Several studies have demonstrated the association between diet and MetS. We performed a systematic review and meta-analysis to provide an estimate of the association between dietary patterns defined through a posteriori methods and MetS. A literature search on PubMed, Web of Science, and Scopus databases, up to March 2019, was conducted to identify all eligible case-control, prospective, or cross-sectional studies involving adult subjects of both sexes. Random-effects models were used. Heterogeneity and publication bias were evaluated. Stratified analyses were conducted on study characteristics. Forty observational studies were included in the meta-analysis, which identified the “Healthy” and the “Meat/Western” dietary patterns. The “Healthy” pattern was associated with reduced MetS risk (OR = 0.85; 95% confidence interval (CI): 0.79–0.91) and significantly decreased the risk in both sexes and in Eastern countries, particularly in Asia. Adherence to the “Meat/Western” pattern increased MetS risk (OR = 1.19; 95% CI: 1.09–1.29) and the association persisted in the stratified analysis by geographic area (Asia, Europe, America) and study design. Lifestyle is linked to risk of developing MetS. The “Healthy” and “Meat/Western” patterns are significantly associated with reduced and increased MetS risk, respectively. Nutrition represents an important modifiable factor affecting MetS risk.

## 1. Introduction

Metabolic Syndrome (MetS) has become a relevant public health concern [1] because of its increased prevalence partially explained by aging population and lifestyle factors, including diet [2,3].

MetS is a pathophysiological state and a cluster of interrelated factors including abdominal obesity, insulin resistance, dysglycemia, hypertension, and dyslipidemia (triglycerides and HDL-C—high-density lipoprotein cholesterol) [4]. The diagnosis of MetS requires three or more of the following criteria: (i) waist circumference >102 cm in men and >88 cm in women; (ii) HDL-C <40 mg/dL (<1.04 mmol/L) in men and <50 mg/dL (<1.29 mmol/L) in women; (iii) triglycerides ≥150 mg/dL (≥1.7 mmol/L); (iv) blood pressure ≥130/85 mmHg and (v) fasting glucose ≥110 mg/dL (≥6.1 mmol/L) [4,5]. A harmonization of the diagnostic criteria has been proposed, as the reference thresholds for abdominal obesity vary considerably among countries and international organizations [4]. In particular, the recommended waist circumference cutoff points are lower for both men and women in Asia, Sub-Saharan Africa, and Central and South America [4].

According to literature, the consumption of specific foods or nutrients is strongly related to the risk of developing MetS [6,7,8,9]. Nutritional epidemiology currently applies dietary patterns to analyze the relation of diet with chronic diseases rather than focusing on individual foods and nutrients [10,11]. Dietary patterns provide a closer representation of the overall dietary habits of the population in study. The statistical methods identifying dietary patterns are distinguished in a priori and a posteriori methods. A priori approaches assign dietary indices and scores (i.e., glycemic index, Mediterranean score) based on current nutritional knowledge of positive and negative effects of various nutrients or foods and identify an optimal pattern, the adherence to which could maximize health benefit. The a priori approach can prove more advantageous only if important dietary factors have been clearly defined to affect the outcome under study [10,12]. Conversely, a posteriori methods identify dietary patterns (i.e., Western and Healthy patterns) based on available dietary data directly obtained from the studied population [10]. Their major limit is that the identified dietary pattern may be sample specific and influenced by subjective decisions [10,12]. The association of MetS outcomes with a priori patterns, such as the Mediterranean diet and inflammatory diet, have been analyzed. The Mediterranean diet reduced the risk of MetS, whereas the comparison of the most pro-inflammatory diet versus the most anti-inflammatory diet showed no significant association [13,14]. A recent meta-analysis [15] had evaluated the relationship between a posteriori dietary patterns and MetS and showed a risk reduction of 11% for prudent/healthy pattern and a risk increase of 16% for Western/unhealthy pattern. A previous meta-analysis [16], found that an inverse association of prudent/healthy pattern and a positive association of Western/unhealthy pattern with MetS in cross-sectional studies, but not in cohort studies. Since then several other studies have been published on this topic with contrasting results. Therefore, we conducted a meta-analysis for deriving a more precise estimation of this association.

The aim of our systematic review and meta-analysis is to investigate and provide an estimate of the association between dietary patterns defined by a posteriori methods and MetS risk in adults.

## 2. Materials and Methods

The present meta-analysis was conducted following the MOOSE (Meta-analysis of Observational Studies in Epidemiology) guidelines [17] and PRISMA (Preferred Reporting Items for Systematic reviews and Meta-Analyses) statement [18].

### 2.1. Search Strategy and Data Source

We conducted a comprehensive literature search, without restrictions, up to 31 March 2019 through PubMed (http://www.ncbi.nlm.nih.gov/pubmed/), Web of Science (http://wokinfo.com/) and Scopus (https://www.scopus.com/) databases to identify all the original articles on the association between dietary patterns and MetS. The literature search included the following search medical subject headings (MeSH) and key words: (“Metabolic Syndrome” OR MetS) AND (“dietary pattern” OR “eating pattern” OR “food pattern” OR “dietary habit” OR “dietary score” OR “dietary index” OR "nutrient pattern” OR “diet diversity” OR “diet variety” OR “diet quality” OR “diet index” OR “diet score”) AND (“factor analysis” OR “principal component analysis” OR “cluster analysis” OR clustering OR “reduced rank regression” OR “data-driven approach” OR “a posteriori method”).

We manually examined the reference lists of selected articles and recent relevant reviews to identify possible additional relevant publications.

### 2.2. Eligibility Criteria

Articles were included if they met the following criteria: (i) evaluated the relationship between dietary patterns derived by a posteriori methods, such as principal component analysis (PCA), factor analysis (FA), and principal component factor analysis (PCFA), and by reduced rank regression (RRR, i.e., an integration of the a priori and the a posteriori approaches) and MetS in adults; (ii) used a case-control, prospective or cross-sectional study design; (iii) reported odds ratio (OR), relative risk (RR) or hazard ratio (HR) estimates with 95% confidence intervals (CIs). For each potentially included study, two investigators independently carried out the selection, data abstraction, and quality assessment. Disagreements were resolved by discussion or in consultation with the third author. Although useful to have background information, reviews and meta-analysis were excluded. No studies were excluded for weakness of design or data quality.

### 2.3. Data Extraction and Quality Assessment

For each selected study, we extracted the following information: first author’s last name, year of publication, country, study design, sample size (when possible, number of cases and controls; cohort size and incident cases), population characteristics (sex, age), duration of follow-up for cohort studies, MetS assessment method, dietary assessment and dietary pattern identification methods (FA, PCA and PCFA), characteristics of the dietary assessment method, name given to the dietary patterns and their characteristics, cutoff points of the different categories of adherence to the dietary pattern (dichotomy, tertile, quartile and quintile), risk estimates with 95% CIs for the different categories of adherence, *p*-value for trend, and confounding factors adjustment. When multiple estimates were reported in the article, we pulled out those adjusted for the most confounding factors.

### 2.4. Statistical Analysis

The estimated overall effect-size statistic was the average of the logarithm of the observed OR (approximated to RR, when necessary) associated with the highest versus the lowest level of adherence to the different dietary patterns. We used the random-effects model to calculate the summary OR and 95% CIs. We restricted the analysis to the dietary patterns defined a posteriori. Since the labeling of the patterns is arbitrary and the dietary patterns are population-specific, we considered only those patterns sharing most foods with similar factor loadings. For the inclusion in the meta-analysis, the two most common dietary patterns with similar factor loading of principle components were identified in 38 studies (out of 40) [19,20,21,22,23,24,25,26,27,28,29,30,31,32,33,34,35,36,37,38,39,40,41,42,43,44,45,46,47,48,49,50,51,52,53,54,55,56,57,58]. The first dietary pattern, named “Healthy”, was characterized by a high loading of vegetables and fruit, poultry, fish, and whole grains. The selected articles labeled this pattern as “Healthy” [22,24,26,27,32,36,43,51,52,54,55,58], “Healthy Japanese” [35], “Health-conscious” [44], “Prudent” [28,31,33,37,46,47,50], “Balanced” [19,25], “Fruit & vegetables” [20,57], “Vegetables, fruits, cereals, and tubers” [42], “Traditional Chinese” [56], “Minimally processed/processed” [21], “Mixed-traditional” [23], “Fruits, vegetables, nuts, and legumes” [29], “Refined Grains & Vegetables” [30], “Traditional” [34,49], “Traditional Lebanese” [38], “Balanced Korean” [39], “Fruit and dairy” [40], “Grains, vegetables, and fish” [45].

The second dietary pattern, named “Meat/Western”, had a high loading of red meat, processed meat, animal fat, eggs and sweets. The included articles labeled this pattern as “Western” [19,20,28,31,43,46,50,51,53,54,58], “Traditional and protein” [42], “Unhealthy” [36,55], “Animal food” [56], “Common Brazilian meal” [57], “Ultra-processed” [21], “Westernized” [24,32], “Mixed-modern” [23], “High-protein/cholesterol” [25], “Meat” [26,34], “Refined and Processed” [27], “Animal protein” [29], “Organ Meat & Poultry” [30], “Fat, meat and alcohol” [32], “High-fat/Western” [33], “Animal food” [35], “Southern” [37], “High-Protein” [38], “Semi-Western” [39], “Alcohol and meat” [40,45], “Processed foods” [44], “High-protein/fat” [47], “Meat and French fries” [49], “High glycemic index and high-fat” [52].

The chi-square-based Cochran’s Q statistic and the I^2^ statistic were used to evaluate heterogeneity in results across studies [59]. The I^2^ statistic yields results ranged from 0% to 100% (*I*^2^ = 0%–25%, no heterogeneity; *I*^2^ = 25%–50%, moderate heterogeneity; *I*^2^ = 50%–75%, large heterogeneity; and *I*^2^ = 75%–100%, extreme heterogeneity) [60]. Results of the meta-analysis may be biased if the probability of publication is dependent on the study results. We used the methods of Begg and Mazumdar [61] and Egger et al. [62] to detect publication bias. Both methods tested for funnel plot asymmetry, the former being based on the rank correlation between the effect estimates and their sampling variances, and the latter on a linear regression of a standard normal deviate on its precision. If a potential bias was detected, we further conducted a sensitivity analysis to assess the robustness of combined effect estimates, and the possible influence of the bias, and to have the bias corrected. We also conducted a sensitivity analysis to investigate the influence of a single study on the overall risk estimate, by omitting one study in each turn. We considered the funnel plot to be asymmetrical, if the intercept of Egger’s regression line deviated from zero, with a *p*-value of <0.05. The analyses were performed using the ProMeta Version 3.0 statistical program (Internovi, Via Cervese, 47522, Cesena, Italy).

## 3. Results

### 3.1. Study Selection

**Figure 1:**
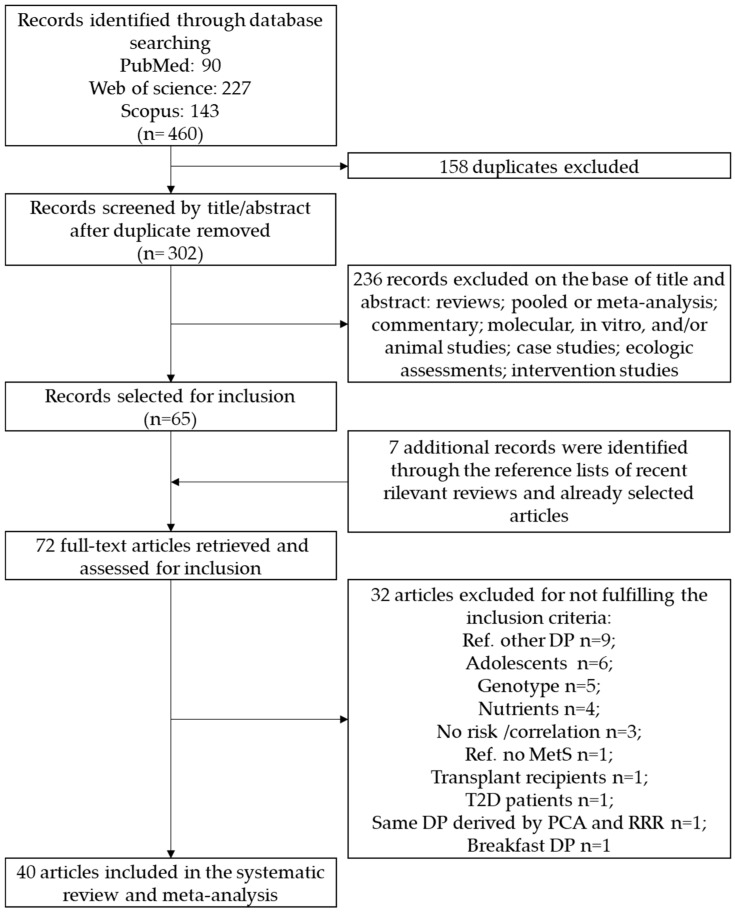
Flow diagram of the systematic literature search on dietary patterns and MetS risk. Metabolic Syndrome (MetS).

The primary literature search through PubMed (*n* = 90), Web of Science (*n* = 227) and Scopus (*n* = 143) databases identified a total of 460 articles. Duplicates (*n* = 158) were removed and the remaining 302 records were identified for title and abstract revision (Figure 1).

Among these, 236 articles were excluded as not investigating the association between dietary patterns and the outcome of interest. Sixty-five articles were subjected to full-text revision. Hand searching of reference lists of both selected articles and recent relevant reviews led to the identification of seven additional items. Subsequently, 32 papers were excluded because they did not meet the inclusion criteria as follows: 9 studies considered a different dietary pattern as the comparison reference; 6 studies were carried out on adolescents; 5 studies reported the MetS risk combined with genotype; 4 studies derived the dietary patterns considering nutrients instead of food items; 3 studied reported the correlation instead of risk estimate; one study used a control group (no MetS) as reference; one study was carried out on transplant recipients; and one study was carried out on type 2 diabetes. Therefore, at the end of the selection process, 40 studies were enclosed for the identification of the different dietary patterns in the systematic review and meta-analysis [19,20,21,22,23,24,25,26,27,28,29,30,31,32,33,34,35,36,37,38,39,40,41,42,43,44,45,46,47,48,49,50,51,52,53,54,55,56,57,58].

### 3.2. Study Characteristics and Quality Assessment

General characteristics of the 40 studies evaluating the association between adherence to a posteriori dietary patterns with MetS risk are shown in Table 1.

These studies were published between 2007 and 2019. Eight studies were conducted in Korea [24,28,34,36,39,40,43,45], eight in Europe [20,22,31,32,41,44,52,53]; six in Iran [19,46,51,54,55,58]; four in the USA [29,37,49,50]; three in China [25,30,56]; two in Japan [33,35], Brazil [42,57], Samoan Islands [23,48] and Lebanon [21,38]; and one each in Thailand [26], Australia [27] and Mexico [47]. Four were cohort studies [36,50,53,54], one was a case-control study [25] and all others were cross-sectional studies. Six studies were conducted on women and men separately [24,26,30,34,39,41], three were on women only [28,43,51] and all others estimated the MetS risk on women and men together. One study did not report the parameters used to identify the MetS [29].

Thirty-one studies used a food frequency questionnaire (FFQ; 43 to 168 items) [19,20,21,22,23,25,26,28,29,31,32,33,34,36,37,38,42,43,46,47,48,49,50,51,52,53,54,55,56,57,58] while six studies used a 24-h dietary recall [24,27,30,39,40,45] to collect dietary information. In addition, three studies used a diet history questionnaire [35], 3-day food diary [41] and 4 weeks face-to-face dietary history interview [44], respectively. One study [53] derived dietary patterns through RRR, another study [48] used a “partial least squares regression” method, while all the other studies derived dietary patterns through a posteriori methods (PCA, PCFA, and FA). Nine studies [21,31,36,41,44,51,52,53,55] reported the association of MetS risk with two different dietary patterns, 24 studies [19,20,22,23,24,25,26,27,28,29,34,35,37,38,39,42,43,47,48,49,50,54,56,58] considered three different dietary patterns, six studies [30,32,33,40,45,57] considered four different dietary patterns and one study [46] considered five different dietary patterns.

### 3.3. Meta-Analysis

**Figure 2:**
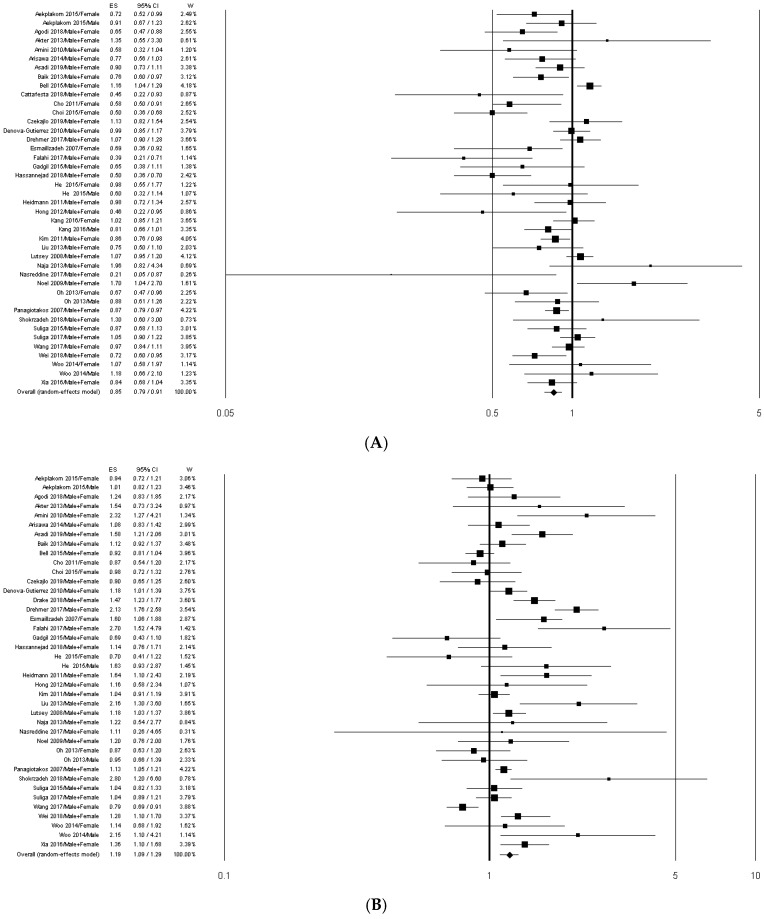
Forest plots of the association between “Healthy” (**A**) and “Meat/Western” (**B**) dietary patterns and MetS risk. ES, effect size.

We identified two common dietary patterns with similar factor loading of principle components: “Healthy” and “Meat/Western” patterns. Thirty-eight out of 40 articles included in the systematic review were used for the overall risk estimation. Two studies [41,48] were excluded because they reported dietary patterns that could not be clearly assumed in “Healthy” nor in “Meat/Western” patterns. In the studies by Agodi et al. [31] and by Wang et al. [23], the “Healthy” dietary pattern was the only pattern identified, whereas in the study by Cattafesta et al. [42] the “Meat/Western” was the only pattern selected. The meta-analyses on the MetS risk in association with “Healthy” and “Meat/Western” dietary patterns (studies comparing the highest intake to the lowest intake) are shown in Figure 2A,B, respectively. 

The overall analysis showed that the MetS risk significantly decreased in association with the adherence to the “Healthy” pattern (OR = 0.85; 95% CI: 0.79–0.91) and significantly increased in association with the adherence to the “Meat/Western” pattern (OR = 1.19; 95% CI: 1.09–1.29). These results did not essentially change when the studies [27,33,52] not comparing the highest vs. the lowest dietary pattern adherence values were excluded (Table 2).

In the “Healthy” pattern meta-analysis, the stratification by study design showed a significant reduced MetS risk in the cross-sectional studies only (Table 2). Stratifying the analysis by geographic area, MetS risk decreased significantly in Eastern countries (OR = 0.78; 95% CI: 0.71–0.86), particularly in Asia (OR = 0.77; 95% CI: 0.70–0.85). The preventive effect of the “Healthy” pattern resulted statistically significant in both sexes (Table 2). 

In the “Meat/Western” pattern meta-analysis, the stratification by study design showed a significantly higher MetS risk in both cohort and cross-sectional studies (Table 2). Similarly, when stratifying the analysis by the geographic area the MetS risk significantly increased in Asia, America and Europe, and in Eastern and Western countries (Table 2). No significant association was found when stratifying by sex (Table 2).

The high heterogeneity in the pooled analysis of both “Healthy” and “Meat/Western” patterns was slightly reduced in the stratification by geographic area.

Sensitivity analyses suggested that the estimates were not substantially modified by any single study. Small changes were found in the risk estimates after removal of the outlier studies by Naja et al. [38] (OR = 0.84; 95% CI: 0.78–0.91) and by Nasreddine et al. [21] (OR = 0.85; 95% CI: 0.79–0.92) in the “Healthy” pattern analysis, and by Shokrzadeh et al. [55] (OR = 1.20; 95% CI: 1.09–1.32) and by Gadgil et al. [29] (OR = 1.23; 95% CI: 1.11–1.35) in the “Meat/Western” pattern analysis.

In the meta-analysis on the “Healthy” pattern, a significant publication bias was detected by the Egger’s test in the overall analysis (*p* = 0.005) and in cross-sectional studies (*p* = 0.016), but not by the Begg’s method (Table 2). In the analysis performed excluding the studies by Bell et al. [27], by Arisawa et al. [33] and by Panagiotakos et al. [52], the publication bias, although reduced, remained significant (*p* = 0.011) (Table 2). In the meta-analysis on “Meat/Western” pattern, a significant publication bias was detected by Egger’s method in the Eastern countries (*p* = 0.021) and by the Begg’s test in men (*p* = 0.042) (Table 2).

**Figure 3:**
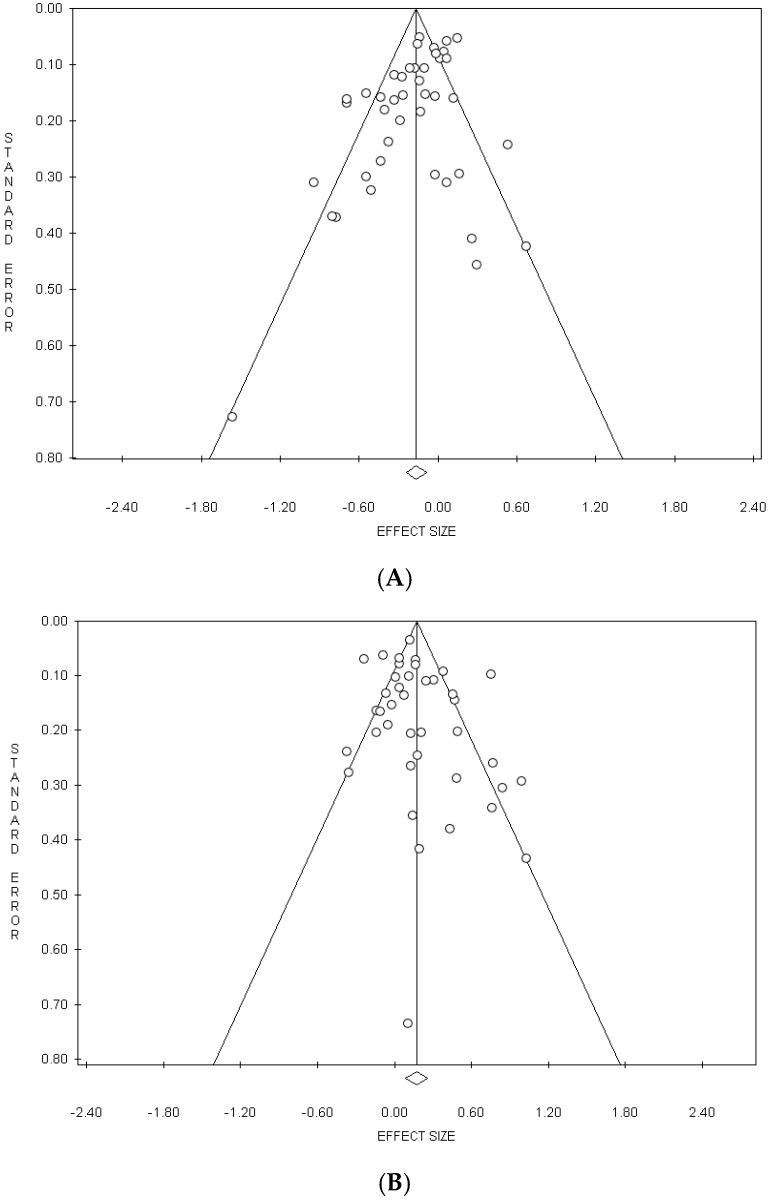
Funnel plots of the meta-analyses on the Healthy” (**A**) and “Meat/Western” (**B**) dietary patterns.

The funnel plots of the meta-analyses on the “Healthy” pattern and on the “Meat/Western” pattern are shown in Figure 3A,B, respectively.

## 4. Discussion

Our systematic review and meta-analysis investigated the effect of dietary patterns extracted via a posteriori methods on MetS risk. According to literature, several different health outcomes are associated with unhealthy and healthy dietary patterns. In particular, the Western/unhealthy pattern increases the risk of cancer in different sites [63,64,65,66,67,68] and the risk of low bone mineral density and osteoporotic fracture [69]. Moreover, the prudent/healthy pattern is associated with lower risk of cardiovascular disease and coronary heart disease [70], diabetes mellitus [71,72], and cognitive decline and dementia [73].

Considering the 40 included articles, we identified two prevalent dietary patterns: “Healthy” and “Meat/Western”. The “Healthy” pattern was associated with a lower MetS risk and significantly decreased the risk in both sexes and in Eastern countries, particularly in Asia. Adherence to the “Meat/Western” pattern was positively associated with MetS risk and this association persisted in the stratified analysis by geographic area and study design. Similarly, the recent meta-analyses by Shab–Bidar et al. [15] and Rodríguez–Monforte et al. [16] showed that a Western/unhealthy pattern significantly increased MetS risk, whereas a prudent/healthy pattern significantly lowered MetS risk. In our study, MetS risk through unhealthy dietary patterns increased by 19%, while it increased by 22% in the study by Shab–Bidar et al. [15] and by 28% in the study by Rodríguez–Monforte et al. [16]. Healthy dietary patterns significantly decreased MetS risk by 15% in our analysis, by 11% in the meta-analysis by Shab–Bidar et al. [15] and by 17% in the meta-analysis by Rodríguez–Monforte et al. [16]. It should be noted that the meta-analysis of Shab–Bidar et al. [15] was performed on cross-sectional studies only and that Rodríguez–Monforte et al. [16] selected 31 studies including those which identified the dietary patterns via cluster analysis (a priori method).

According to our findings, the “Meat/Western” pattern significantly increased MetS risk of 20% in Asia, 15% in Europe and 33% in America. In dietary patterns derived a posteriori, the factor loadings indicate the most commonly consumed foods, reflecting the cultural influence on food consumption [74,75]. It is noteworthy that the usual diet of European populations, especially in Mediterranean countries, tend to include the consumption of healthy foods, such as seafood, vegetables, and fruit, whereas American populations mostly adhere to Westernized dietary patterns, containing high pro-inflammatory foods [76]. As reported in the study by Calton et al. [77], other pre-defined representative dietary patterns exist worldwide, such as the Dietary Approaches to Stop Hypertension (DASH) diet, which is characterized by high intake of fruit, vegetables, whole grains and dairy [78], and the Northern Europe dietary pattern, which is characterized by high intake of fruit, vegetables, legumes, low-fat dairy, fatty fish, oats, barley and almonds [79]. These patterns can affect MetS risk and should be evaluated when investigating the effect of the dietary patterns on developing MetS, as culture and society influence adherence to healthy or unhealthy dietary pattern [77]. Our study combined dietary patterns derived a posteriori from world countries with very different eating habits, in particular, traditional dietary patterns from Eastern Asian countries (Japan [33,35], China [25,30,56], Korea [24,28,34,36,39,40,43,45]), from Western Asian countries (Iran [19,46,51,54,55,58]), from the Mediterranean area (Greece [52], Lebanon [21,38]), from Northern Europe (Sweden [53]), from Middle Europe (Germany [44], Czech Republic [31] and Poland [20,22,32]), from North America (USA [29,37,49,50]), from South America (Brazil [42,57]), and from Australia [27]. Indeed, the traditional dietary pattern in Asian countries is characterized by high intake of rice and/or kimchi, fish and sea food, soybean and soybean products, mushrooms, vegetables, and fruit [24,28,34,39,40,43,56], in Poland by red meat, fish, potatoes, soup, refined grains and sugars, and high-fat milk [20,22], and in Iran by refined grains, nuts, eggs, vegetables and legumes, potatoes, and hydrogenated fats [51,58].

Despite the influence of sex-related factors on MetS [80], we observed no sex-related difference on the association of dietary pattern with MetS, but, notably, the “Healthy” pattern showed a stronger protective effect in women. 

The “Meat/Western” pattern, characterized by high intake or red and processed meat, eggs, refined grains, and sweets, resulted associated with an increased (+19%) MetS risk. These foods plausibly represent the main cause of the observed effect on MetS risk, particularly meat [81,82], since refined carbohydrates, red and processed meats, and fried foods have pro-inflammatory properties and can increase inflammatory cytokines [83]. Indeed, although the meta-analysis by Namazi et al. [14] found no significant association between the most pro-inflammatory diet and MetS, inflammatory factors are involved in insulin resistance and lipid disorders [83].

Our results showed the association of the “Healthy” pattern with a lower (−15%) MetS risk. The healthy patterns are characterized by the consumption of foods with high content of vitamins, minerals, antioxidants, fiber, MUFA and n-3 fatty acids, which could contribute to explain the protective effect of the “Healthy” pattern on MetS. Indeed, higher adherence to healthy dietary patterns is associated with a lower risk of glucose intolerance, weight gain, inflammation, insulin resistance and a higher level of HDL cholesterol [84].

### Limitations

The main limitation of our study is that the risk of developing MetS could be associated with dietary patterns other than the two (“Healthy” and “Meat/Western”) discussed in this meta-analysis. Differences in the populations in study and in the referral values for MetS diagnosis represent another study limitation and result in heterogeneity. Indeed, the high heterogeneity may be related to the wide variability in dietary data collection and analysis, in the various and not uniformly adjusted confounding factors, and in the identification of the dietary patterns. Heterogeneity is more evident in the analysis on “Meat/Western” pattern, as a possible consequence of the difficulty in characterizing this pattern across the selected studies. Another limitation is that pooled data were directly driven by the included studies, presenting their own weaknesses in study design. Moreover, the cross-sectional nature of many included studies precludes causal inference and the dietary pattern may represent a *post hoc* event. Only the OR of the highest and the lowest quantile of healthy or unhealthy dietary patterns were included in our analysis, limiting the evaluation of the presence of any trend. Finally, some studies reported risk estimates for quintiles, others for quartiles, and others for tertiles. As dietary intakes are influenced by sex, race/ethnicity, and societal factors, our findings should be considered in the different geographic contexts. Thus, these aspects may have affected the reproducibility of the association between dietary patterns and MetS.

To further advance this field of research, future studies are needed to examine the association between dietary patterns in geographic context not yet described and MetS, and to evaluate the impact of dietary patterns on the determinants of MetS.

## 5. Conclusions

A protective effect on MetS is attributed to adherence to the “Healthy” pattern, which is characterized by high consumption of fruit, vegetables, whole grains, poultry, fish, nuts, legumes, and low-fat dairy products, whereas the “Meat/Western” pattern is positively associated with MetS. Nutrition is one of the most important modifiable factors affecting health. Public health efforts should aim to adopt healthy dietary patterns and to reduce the burden of MetS, providing guidance for nutritional intervention. For further advance in research, more prospective studies are needed to investigate the association between dietary patterns and MetS in each gender and in different geographic context.

## Figures and Tables

**Table 1:** Main characteristics of studies included in the systematic review and meta-analysis on dietary patterns and Metabolic Syndrome.

First Author Year Location	Study Design, Name, and Population Cases/Controls Follow-Up Incident Cases Age	Assessment of Metabolic Syndrome	Dietary Pattern Assessment and Identification Method	Dietary Pattern Type and Characteristics	Pattern Score	OR/RR (95% CI)	*p* for Trend	Matched or Adjusted Variables
Asadi [19] 2019 Iran	Cross-sectional Mashhad stroke and heart atherosclerotic disorder (MASHAD) study Cases: 1890 Age 50.11 ± 7.76 Control: 4005 Age 47.56 ± 8.21	- WC^1^: ≥94 cm (men); ≥80 cm (women); - SBP/DBP^2^: ≥130/85 mmHg; - FBG^3^: ≥100 mg/dL; - TG^4^: ≥150 mg/dL; - HDL-c^5^: <40 mg/dL (men); <50 mg/dL (women)	65-item FFQ^6^ (IA^7^) 22 food groups FA^8^ Varimax rotation EIG^9^>1 3 factors VE^10^ 23%	***1. Balanced***: vegetables, green leafy vegetables, fruit, dairy products, red meats, poultry, legumes	Tertile 1 Tertile 3	1.00 (Reference) 0.90 (0.73–1.11)	0.343	Age, sex, BMI^11^, physical activity, smoking, education, marital status, total energy intake
				***2. Western***: sugar, tea, eggs, potato, snacks, organs meat, nuts, butter, pickled foods, carbonated beverages, red meats	Tertile 1 Tertile 3	1.00 (Reference) 1.58 (1.21–2.06)	0.001	
				***3. High-carbohydrate***: refined grains, carbonated beverages, fast foods, snacks, sugar, coffee, sea foods	Tertile 1 Tertile 3	1.00 (Reference) 1.17 (1.02–1.33)	0.023	
Czekajlo [20] 2019 Poland	Cross-sectional Prospective Urban and Rural Epidemiological (PURE) study Cases: 721 Age 56.4 ± 9.1 Control: 913 Age 53.0 ± 10.1	- WC: ≥94 cm (men); ≥80 cm (women); - SBP/DBP: ≥130/85 mmHg; - FBG: ≥100 mg/dL; - TG: ≥150 mg/dL; - HDL-c: <40 mg/dL (men); <50 mg/dL (women)	154-item FFQ 22 food groups PCA^12^ Varimax rotation Loading >0.5 3 factors VE 35.2%	***1. Western***: refined grains, processed meat, sweets and sugar, honey	Quartile 1 Quartile 4	1.00 (Reference) 0.90 (0.65–1.25)		Age, sex, residence, education, physical activity, smoking, total energy intake
				***2. Fruit and vegetables***: fruit, vegetables and nuts, seeds and raisins	Quartile 1 Quartile 4	1.00 (Reference) 1.13 (0.82–1.54)		
				***3. Traditional***: mixed dishes, soups, fish and red meat	Quartile 1 Quartile 4	1.00 (Reference) 1.28 (0.95–1.72)		
Agodi [31] 2018 Czech Republic	Cross-sectional Kardiovize Brno 2030 study Cases: 739 Age 54.0 (IQR 15) Control: 1195 Age 41.5 (IQR 17)	- WC: ≥ 94 cm (men); ≥80 cm (women); - SBP/DBP: ≥130/85 mmHg; - FBG: ≥100 mg/dL; - TG: ≥150 mg/dL; - HDL-c: <40 mg/dL (men); <50 mg/dL (women)	43-item FFQ 31 food groups PCA Varimax rotation EIG>2 Loading ≥0.25 2 factors VE 13.73%	***1. Western***: white bread, processed meat, fries, hamburger	Tertile 1 Tertile 3	1.00 (Reference) 1.24 (0.83–1.85)	0.132	Age, sex, marital status, employment, education, smoking, BMI, total energy intake, physical activity
				***2. Prudent***: cereals, jam and honey, fish, fruit	Tertile 1 Tertile 3	1.00 (Reference) 0.65 (0.47–0.88)	0.004	
Cattafesta [42] 2018 Brazil	Cross-sectional 515 bankers Age 20–64 Cases: 85 Control: 410	- WC: >102 cm (men); >88 cm (women); - SBP/DBP: ≥130/85 mmHg; - FBG: ≥100 mg/dL; - TG: ≥150 mg/dL; - HDL-c: <40 mg/dL (men); <50 mg/dL (women)	73-item FFQ (IA) PCA Varimax rotation Loading >0.3 3 factors	***1. Vegetables, fruit, cereals, and tubers***: cabbage, carrot, cucumber, pumpkin, zucchini, okra, chayote, cauliflower, beet and pod, lettuce, tomato, papaya, apple, pear, watermelon, guava, mango, pineapple, grape, orange, manioc, polenta, cooked potatoes, onion, garlic, peppers	Quintile 1 Quintile 3 Quintile 5	1.00 (Reference) 0.305 (0.138–0.672) 0.447 (0.216–0.926)	0.003	NR^13^
				***2. Sweets and snacks***: lentils, cake, ice cream, chocolate, pudding, chocolate powder, pizza, salty fish, canned fish and shrimp, wine, viscera, and avocado		NR		
				***3. Traditional and protein***: rice, beans, pork, bone-in beef and beef steak, sausage, eggs, potato chips, hamburger, bacon, mayonnaise, sweet bread, salt bread, butter/margarine		NR		
Drake [53] 2018 Sweden	Cohort study Malmö Diet and Cancer Study (MDCS) 2368 subjects Age 45–67 follow-up: 16.7 1131 incident cases	- WC: >102 cm (men); >88 cm (women); - SBP/DBP: ≥130/85 mmHg; - FBG: ≥100 mg/dL; - TG: ≥150 mg/dL; - HDL-c: <40 mg/dL (men); <50 mg/dL (women)	1. 7-d food record 2. 168-items FFQ 3. Diet history interview 38 food groups RRR^14^ 2 factors VE 3.2%	***1. Western***: sugar-sweetened beverages, milk (reduced fat), artificially sweetened beverages, red and processed meat, sweets	Quartile 1 Quartile 4	1.00 (Reference) 1.47 (1.23–1.77)	<0.001	Age, sex, total energy intake, height, smoking, education, total physical activity, co-habiting status
				***2. Drinker***: alcoholic beverages, red and processed meat, fish and shellfish, eggs	Quartile 1 Quartile 4	1.00 (Reference) 1.00 (0.85–1.19)	0.88	
Hassannejad [54] 2018 Iran	Cohort study Isfahan Cohort Study (ICS) 1387 participants follow-up: 13	- WC: >102 cm (men); >88 cm (women); - SBP/DBP: ≥130/85 mmHg; - FBG: ≥100 mg/dL; - TG: ≥150 mg/dL; - HDL-c: <40 mg/dL (men); <50 mg/dL (women)	48-item FFQ (IA) 21 food groups PCA Varimax rotation EIG>1.5 loading >0.2 3 factors VE 26.2%	***1. Healthy***: fruit, vegetables, olive oils, chicken, fish, nuts and beans	2 categories	1.00 (Reference) 0.50 (0.36–0.70)		Age, sex, socioeconomic status, smoking, physical activity, BMI, medications for hypertension and diabetes
				***2. Iranian***: dairy product, animal fat, sweets, organ meat, red meat and hydrogenated oils	2 categories	1.00 (Reference) 1.28 (1.01–1.65)		
				***3. Western***: fried foods, rice, red meat, hydrogenated oils, carbonated beverages, fast foods, canned food, sweets	2 categories	1.00 (Reference) 1.14 (0.76–1.71)		
Shokrzadeh [55] 2018 Iran	Cross-sectional 304 men and women, Age 20–60	- WC: >102 cm (men); >88 cm (women); - SBP/DBP: ≥130/85 mmHg; - FBG: ≥5.6 mmol/L; - TG: ≥1.7 mmol/L; - HDL-c: <1.03 mmol/L (men); <1.29 mmol/L (women)	147-item FFQ (IA) 24 food groups FA 2 factors VE 18.3	***1. Healthy***: fruit, vegetables, olive, nuts, legumes, cereal, low-fat dairy products, liquid oil, olive oil, fish	Tertile 1 Tertile 3	1.00 (Reference) 1.3 (0.6–3.0)	0.55	Age, sex, physical activity
				***2. Unhealthy***: snacks, red meat, fat dairy, mayonnaise, tuna, organ meats, processed meats, sweets, pizza, spices, ketchup	Tertile 1 Tertile 3	1.00 (Reference) 2.8 (1.2–6.6)	0.09	
Wei [56] 2018 China	Cross-sectional 1918 individuals Age 45–59 Cases: 453 Age 54.82 ± 9.63 Control: 1465 51.48 ± 9.56	- WC: ≥90 cm (men); ≥85 cm (women); - SBP/DBP: ≥130/85 mmHg; - FBG: ≥5.6 mmol/L; - TG: ≥1.7 mmol/L; - HDL-c: <1.0 mmol/L (men); <1.3 mmol/L (women)	138-item FFQ (IA) 30 food groups PCFA^16^ Varimax rotation EIG≥1.5 Loading ≥0.4 3 factors VE 23%	***1. Traditional Chinese***: whole grains, tubers, vegetables, fruit, pickled vegetables, mushrooms, bacon, salted fish, salted and preserved eggs, soya bean and its products, miscellaneous beans, vegetable oil, tea	Quartile 1 Quartile 4	1.00 (Reference) 0.72 (0.596–0.952)	<0.05	Sage, sex, education, physical activity, smoking, total energy intake
				***2. Animal food***: red meats, poultry and organs, processed and cooked meat, fish and shrimp, eggs, seafood, alcoholic beverages, coffee	Quartile 1 Quartile 4	1.00 (Reference) 1.28 (1.103–1.697)	<0.05	
				***3. High-energy***: refined grains, milk, cheese, fats, fast foods, nuts, snacks, chocolates, honey, drinks	Quartile 1 Quartile 4	1.00 (Reference) 1.09 (0.825–1.495)	0.44	
Drehmer [57] 2017 Brazil	Cross-sectional Brazilian Longitudinal Study of Adult Health (ELSA—Brazil) 9835 participants Age 50.7 ± 8.7	- WC: ≥102 cm (men); ≥88 cm (women) SBP/DBP: ≥130/85 mmHg; - FBG: ≥5.6 mmol/L; - TG: ≥1.69 mmol/L; - HDL-c: <1.03 mmol/L (men); <1.29 mmol/L (women)	114-item FFQ PCA Varimax rotation EIG ≥1.5 Loading ≥0.2 4 factors VE 23%	***1. Vegetables/fruit***: vegetables and fruit	Quintile 1 Quintile 5	1.00 (Reference) 1.07 (0.90–1.28)	0.366	Age, sex, race, education, family income, occupational status, study center, menopausal status, family history of diabetes, BMI, physical activity, smoking, alcohol, calorie intake
				***2. Common Brazilian fast foods/full fat dairy/desserts***: fast foods, cakes, milk-based desserts, regular cheese and red meats	Quintile 1 Quintile 5	1.00 (Reference) 0.86 (0.71–1.04)	0.057	
				***3. Common Brazilian meal***: white rice, beans, beer, processed and fresh meats	Quintile 1 Quintile 5	1.00 (Reference) 2.13 (1.76–2.58)	<0.001	
				***4. Diet or light foods and beverages/low-fat dairy***: low-fat foods, low or zero sugar beverages with artificial sweeteners and low-fat dairy	Quintile 1 Quintile 5	1.00 (Reference) 1.47 (1.23–1.71)	<0.001	
Falahi [58] 2017 Iran	Cross-sectional 973 persons Age 18–75	- WC: ≥102 cm (men); ≥88 cm (women); - SBP/DBP: ≥130/85 mmHg; - FBG: ≥100 mg/dL; - TG: ≥150 mg/dL; - HDL-c: <40 mg/dL (men); <50 mg/dL (women)	168-item FFQ (SA) 40 food groups PCFA Loading >0.2 3 factors VE 29.9%	***1. Western***: red meat, processed meat, organ meats, margarine, coffee, sweets and desserts, soft drinks, condiments, dried fruit	Quintile 1 Quintile 5	1.00 (Reference) 2.70 (1.52–4.79)	0.002	Age, sex, smoking, physical activity, drug using, history of diabetes, history of heart disease, BMI
				***2. Healthy***: poultry, dairy products, fish, fruit, yellow vegetables, cruciferous vegetables, green leafy vegetables, other vegetables, legumes, whole grains, olives	Quintile 1 Quintile 5	1.00 (Reference) 0.39 (0.21–0.71)	0.004	
				***3. Traditional***: grains, tea, nuts, fruit juices, eggs, pickles, hydrogenated oils, vegetables oils, sugar, salt	Quintile 1 Quintile 5	1.00 (Reference) 1.43 (0.80–2.54)	0.48	
Nasreddine [21] 2017 Lebanon	Cross-sectional 302 subjects Cases: 195 Age 43.4 ± 14.7 Controls: 107 Age 37.2 ± 12.9	- WC: ≥94 cm (men); ≥80 cm (women); - SBP/DBP: ≥130/85 mmHg; - FBG: ≥100 mg/dL; - TG: ≥150 mg/dL; - HDL-c: <40 mg/dL (men); <50 mg/dL (women)	80-item FFQ (IA) 25 food groups FA Varimax rotation Loading >0.4 2 factors VE 22.44%	***1. Ultra-processed***: fast foods, snacks, meat, nuts, sweets and liquor	2 categories	1.00 (Reference) 1.11 (0.26–4.65)		Age, sex, marital status, area of residence, education, income, smoking, physical activity, total energy intake, BMI
				***2. Minimally processed/processed***: fruit, vegetables, legumes, breads, cheeses, sugar and fats	2 categories	1.00 (Reference) 0.21 (0.05–0.87)		
Suliga [22] 2017 Poland	Cross-sectional Polish–Norwegian Study (PONS) Study 7997 participants Age 37–66	- WC: ≥94 cm (men); ≥80 cm (women); - SBP/DBP: ≥130/85 mmHg; - FBG: ≥100 mg/dL; - TG: ≥150 mg/dL; - HDL-c: <40 mg/dL (men); <50 mg/dL (women)	67-item FFQ 33 food groups FA Varimax rotation Loading >0.3 3 factors VE 26.7%	***1. Healthy***: fruit and vegetables, sour cabbage, whole grains, yogurt, cottage cheese, fish, nuts	Quartile 1 Quartile 4	1.00 (Reference) 1.05 (0.90–1.22)	0.56	Age, sex, place of living, education, marital status, smoking, physical activity, BMI
				***2. Westernized***: fried dishes, oil, mayonnaise, red meat, processed meat, eggs, sugar-sweetened beverages, alcohol, sugar, sweets	Quartile 1 Quartile 4	1.00 (Reference) 1.04 (0.89–1.21)	0.88	
				***3. Traditional-carbohydrate***: potatoes, refined grains, soups, sugar, sweets, high-fat milk	Quartile 1 Quartile 4	1.00 (Reference) 1.05 (0.90–1.23)	0.593	
Wang [23] 2017 Samoa	Cross-sectional 2774 adults Cases: 1104 Age 49 ± 10 Controls: 1670 Age 42 ± 11	- WC: ≥102 cm (men); ≥88 cm (women); - SBP/DBP: ≥130/85 mmHg; - FBG: ≥100 mg/dL; - TG: ≥1.7 mmol/L; - HDL-c: <1.0 mmol/L (men); <1.3 mmol/L (women)	104-item FFQ 28 food groups PCA Varimax rotation EIG >1.0 Loading ≥0.3 3 factors VE 36%	***1. Modern***: pizza, cheeseburgers, breakfast cereal, margarine, sugary drinks, desserts, snacks, egg products, noodles, nuts, breads, and cakes	Quintile 1 Quintile 5	1.00 (Reference) 1.00 (0.86–1.15)	0.62	Age, sex, material lifestyle score, smoking, total energy intake, physical activity, hypertension medication, diabetes medication
				***2. Mixed-traditional***: fruit, vegetables, soup, poultry, fish, dairy products, breads and cakes	Quintile 1 Quintile 5	1.00 (Reference) 0.97 (0.84–1.11)	0.24	
				***3. Mixed-modern***: red meat, egg products, noodles, **grains**, seafood and coconut products	Quintile 1 Quintile 5	1.00 (Reference) 0.79 (0.69–0.91)	0.006	
Kang [24] 2016 Korea	Cross-sectional KNHANES 5384 men 8026 women Age ≥19	- WC: ≥90 cm (men); ≥80 cm (women); - SBP/DBP: ≥130/85 mmHg; - FBG: ≥100 mg/dL; - TG: ≥150 mg/dL; - HDL-c: <40 mg/dL (men); <50 mg/dL (women)	24-h recall method 24 food groups FA Varimax rotation EIG >1.3 Loading ≥0.25 3 factors VE 20.9% (Men) 20.5% (Women)	***1. Traditional***: white rice and kimchi	***MEN***	***MEN***		Age, BMI, income, smoking, physical activity, educational level, alcohol, energy intake
					Quartile 1	1.00 (Reference)	0.4344	
					Quartile 4	1.08 (0.87–1.35)		
					***WOMEN***	***WOMEN***		
					Quartile 1	1.00 (Reference)	0.0003	
					Quartile 4	1.41 (1.15–1.73)		
				***2. Westernized***: oils, sugar and sweets, vegetables, and fish	***MEN***	***MEN***		
					Quartile 1	1.00 (Reference)		
					Quartile 4	NR		
					***WOMEN***	***WOMEN***		
					Quartile 1	1.00 (Reference)		
					Quartile 4	NR		
				***3. Healthy***: whole grains, legumes, fruit, and seaweed	***MEN***	***MEN***		
					Quartile 1	1.00 (Reference)	0.1341	
					Quartile 4	0.81 (0.66–1.01)		
					***WOMEN***	***WOMEN***		
					Quartile 1	1.00 (Reference)	0.7596	
					Quartile 4	1.02 (0.85–1.21)		
Xia [25] 2016 China	Case-control Tianjin Chronic Low-grade Systemic Inflammation and Health (TCLSI Health) 1636 cases 6677 controls	- WC: ≥90 cm (men); ≥80 cm (women); - SBP/DBP: ≥130/85 mmHg; - FBG: ≥5.56 mmol/L; - TG: ≥1.7 mmol/L; - HDL-c: <1.0 mmol/L (men); <1.3 mmol/L (women)	81-item FFQ FA Varimax rotation EIG >1.0 Loading >0.3 3 factors VE 27.4%	***1. High-carbohydrate/sweet***: candied fruit, cakes, ice cream, and juice	Quartile 1 Quartile 4	1.00 (Reference) 1.04 (0.85–1.28)	0.91	Other dietary pattern factor scores
				***2. Balanced***: balance intake of vegetables, mushroom and coarse cereals	Quartile 1 Quartile 4	1.00 (Reference) 0.84 (0.68–1.04)	0.29	
				***3. High-protein/cholesterol***: animal offal, animal blood, and sausage	Quartile 1 Quartile 4	1.00 (Reference) 1.36 (1.10, 1.68)	<0.01	
Aekplakorn [26] 2015 Thailand	Cross-sectional NHES IV 2693 men 3179 women Age 30–59	- WC: ≥90 cm (men); ≥80 cm (women) - SBP/DBP: ≥130/85 mmHg - FBG: ≥100 mg/dL - TG: ≥150 mg/dL - HDL-c: <40 mg/dL (men); <50 mg/dL (women)	FFQ 22 food groups PCA Varimax rotation EIG >1.5 3 factors VE 32.74% (men) 33.1% (women)	***1. Meat***: red meat, processed meat, and fried food	***MEN***	***MEN***		Age, alcohol drinking, family history of diabetes, smoking, physical activity, BMI
					Quartile 1	1.00 (Reference)		
					Quartile 4	1.01 (0.82–1.23)		
					***WOMEN***	***WOMEN***		
					Quartile 1	1.00 (Reference)		
					Quartile 4	0.94 (0.72–1.21)		
				***2. Healthy***: beans, vegetables, wheat, and dairy products.	***MEN***	***MEN***		
					Quartile 1	1.00 (Reference)		
					Quartile 4	0.91 (0.67–1.23)		
					***WOMEN***	***WOMEN***		
					Quartile 1	1.00 (Reference)		
					Quartile 4	0.72 (0.52–0.99)		
				***3. Carbohydrate***: glutinous rice, fermented fish, chili paste, and bamboo shoots	***MEN***	***MEN***		
					Quartile 1	1.00 (Reference)		
					Quartile 4	1.82 (1.31–2.55)		
					***WOMEN***	***WOMEN***		
					Quartile 1	1.00 (Reference)		
					Quartile 4	1.60 (1.24–2.08)		
Bell [27] 2015 Australia	Cross-sectional 2011–2012 NNPAS 2415 adults Age >45	- WC: ≥102 cm (men); ≥88 cm (women); - SBP/DBP: ≥140/90 mmHg; - FBG: >6.0 mmol/L; - TG: ≥ 2.0 mmol/L; - HDL-c: <1.0 mmol/L (men); <1.3 mmol/L (women)	24-h dietary recall 39 food groups PCFA Varimax rotation EIG >1.5 Loadings > 0.25 3 factors VE 21.9%	***1. Red Meat and Vegetable***: red meat and several types of vegetables	One standard deviation increase	0.99 (0.89–1.10)		
				***2. Refined and Processed***: added sugar, full fat dairy, unsaturated spreads, cakes, pastries, and processed meat		0.92 (0.81–1.04)		
				***3. Healthy***: wholegrains, fresh fruit, dried fruit, legumes and low-fat dairy loaded		1.16 (1.04–1.29)		
Choi [28] 2015 Korea	Cross-sectional 5189 women Age 31–70 mean 52.2 ± 8.3	- WC: ≥80 cm - SBP/DBP: ≥130/85 mmHg - FBG: ≥100 mg/dL - TG: ≥150 mg/dL - HDL-c: <50 mg/dL	106-item FFQ 37 food groups PCA Varimax rotation 3 factors VE 24.7%	***1. Traditional***: vegetables, condiments, shellfish, mushrooms, seaweed, fish, tubers, and kimchi	Quintile 1 Quintile 5	1.00 (Reference) 1.09 (0.83–1.44)	0.44	Age, marital status, education, household income, smoking status, alcohol consumption, regular exercise, and total energy intake.
				***2. Western***: red meat, oil, cake/pizza, noodles, poultry, processed meats, bread, and sweets	Quintile 1 Quintile 5	1.00 (Reference) 0.98 (0.72–1.32)	0.95	
				***3. Prudent***: fruit and fruit products, bread, dairy products, nuts, cake/pizza, and milk	Quintile 1 Quintile 5	1.00 (Reference) 0.50 (0.36–0.68)	<0.001	
Gadgil [29] 2015 USA	Cross-sectional MASALA 892 South Asians Age 40–84	NR	163-item FFQ 29 food groups PCA Varimax rotation EIG >1.0 Loadings > 0.25 3 factors VE 23.2%	***1. Animal protein***: poultry, red meat, eggs, fish	Tertile 1 Tertile 3	1.00 (Reference) 0.69 (0.43–1.10)	0.73	Age, sex, energy intake, study site, income, education, smoking, alcohol intake, exercise, BMI, waist circumference
				***2. Fried snacks, sweets, and high-fat dairy***: butter/ghee, fried snacks, high-fat dairy, potatoes, sweets	Tertile 1 Tertile 3	1.00 (Reference) 0.95 (0.56–1.59)	0.18	
				***3. Fruit, vegetables, nuts, and legumes***: fruit, legumes, nuts, vegetables, vegetables oil	Tertile 1 Tertile 3	1.00 (Reference) 0.65 (0.38–1.11)	0.08	
He [30] 2015 China	Cross-sectional CNNHS Cases: 617 Controls: 1579 Age ≥18	- WC: ≥90 cm (men); ≥80 cm (women) - SBP/DBP: ≥130/85 mmHg - FBG: ≥100 mg/dL - TG: ≥150 mg/dL - HDL-c: <40 mg/dL (men); <50 mg/dL (women)	24-h dietary recall for 3 d FA Varimax rotation EIG >1.0 Loading ≥ 0.5 4 factors VE 48.65%	***1. Refined Grains and Vegetables***: refined grains, vegetables and livestock meat	***MEN***	***MEN***		Age, occupation, types of area, BMI
					Quintile 1	1.00 (Reference)	0.496	
					Quintile 5	0.60 (0.32–1.14)		
					***WOMEN***	***WOMEN***		
					Quintile 1	1.00 (Reference)	0.021	
					Quintile 5	0.98 (0.55–1.77)		
				***2. Dairy and Eggs***: milk, dairy products, eggs, fruit, marine products	***MEN***	***MEN***		
					Quintile 1	1.00 (Reference)	<0.001	
					Quintile 5	1.54 (0.88–2.68)		
					***WOMEN***	***WOMEN***		
					Quintile 1	1.00 (Reference)	0.008	
					Quintile 5	0.45 (0.26–0.79)		
				***3. Organ Meat and Poultry***: organ meat and poultry	***MEN***	***MEN***		
					Quintile 1	1.00 (Reference)	0.087	
					Quintile 5	1.63 (0.93–2.87)		
					***WOMEN***	***WOMEN***		
					Quintile 1	1.00 (Reference)	0.002	
					Quintile 5	0.70 (0.41–1.22)		
				***4. Coarse Grains and Beans***: coarse grain, soybean, bean products	***MEN***	***MEN***		
					Quintile 1	1.00 (Reference)	0.467	
					Quintile 5	0.75 (0.44–1.29)		
					***WOMEN***	***WOMEN***		
					Quintile 1	1.00 (Reference)	0.655	
					Quintile 5	1.35 (0.81–2.22)		
Suliga [32] 2015 Poland	Cross-sectional PONS 2479 subjects with a normal weight Age 37–66	- WC: ≥94 cm (men); ≥80 cm (women); - SBP/DBP: ≥130/85 mmHg; - FBG: ≥100 mg/dL; - TG: ≥150 mg/dL; - HDL-c: <40 mg/dL (men); <50 mg/dL (women)	134-item FFQ 31 food groups PCFA Varimax rotation Loading ≥ 0.3 4 factors VE 32.95%	***1. Healthy***: fruit and vegetables, low-fat milk and dietary products, whole grains food	Tertile 1 Tertile 3	1.00 (Reference) 0.87 (0.68–1.13)		Age, level of education, place of residence, smoking cigarettes and physical activity
				***2. Fat, meat and alcohol***: lard, red meat, cold cured meat, eggs, fried dishes, vegetable oils, mayonnaise and alcoholic drinks	Tertile 1 Tertile 3	1.00 (Reference) 1.04 (0.82–1.33)		
				***3. Prudent***: fish and whole grains products	Tertile 1 Tertile 3	1.00 (Reference) 0.69 (0.53–0.89)		
				***4. Coca-Cola, hard cheese and French fries***: Coca-Cola, hard cheese and French fries	Tertile 1 Tertile 3	1.00 (Reference) 0.82 (0.64–1.04)		
Arisawa [33] 2014 Japan	Cross-sectional J–MICC Cases: 91 Age 53.5 ± 8.9 Controls: 422 Age 51.4 ± 9.4	- WC: ≥90 cm (men); ≥80 cm (women) - SBP/DBP: ≥130/85 mmHg - FBG: ≥100 mg/dL - TG: ≥150 mg/dL - HDL-c: <40 mg/dL (men); <50 mg/dL (women)	46-item FFQ PCA EIG ≥1.0 Loading ≥ 0.2 4 factors VE 33%	***1. Prudent***: fruit, vegetables and mushrooms	One standard deviation increase	0.77 (0.56–1.03)		Age, sex, total energy intake, physical activity, smoking and drinking habits
				***2. High-fat/Western***: meat, meat products, mayonnaise, fried foods, fried dishes, Western-style confectionery		1.08 (0.83–1.42)		
				***3. Bread and dairy***: bread, margarine, mil and yogurt		0.89 (0.69–1.14)		
				***4. Seafood***: squid, shrimp, crab, octopus, shellfish, roe		1.14 (0.91–1.44)		
Woo [34] 2014 Korea	Cross-sectional 486 men 771 women Age 31–70 Cases: 205 Age 55.9 ± 9.2 Controls 1052 Age 50.8 ± 9.0	- WC: ≥90 cm (men); ≥80 cm (women) - SBP/DBP: ≥130/85 mmHg - FBG: ≥100 mg/dL - TG: ≥150 mg/dL - HDL-c: <40 mg/dL (men); <50 mg/dL (women)	103-item FFQ 37 food groups PCA Varimax rotation Loading >0.2 3 factors VE 31.9%	***1. Traditional***: condiments, green/yellow vegetables, light-colored vegetables, tubers, clams, tofu/soymilk, and seaweed	***MEN***	***MEN***		Age, total energy intake, smoking status, alcohol consumption, and physical activity
					Quartile 1	1.00 (Reference)	0.33	
					Quartile 4	1.18 (0.66–2.10)		
					***WOMEN***	***WOMEN***		
					Quartile 1	1.00 (Reference)	0.978	
					Quartile 4	1.07 (0.58–1.97)		
				***2. Meat***: red meat, red meat byproducts, other seafood, and high-fat red meat	***MEN***	***MEN***		
					Quartile 1	1.00 (Reference)	0.005	
					Quartile 4	2.15 (1.10–4.21)		
					***WOMEN***	***WOMEN***		
					Quartile 1	1.00 (Reference)	0.455	
					Quartile 4	1.14 (0.68–1.92)		
				***3. Snack***: cake/pizza, snacks, and bread	***MEN***	***MEN***		
					Quartile 1	1.00 (Reference)	0.335	
					Quartile 4	0.80 (0.49–1.31)		
					***WOMEN***	***WOMEN***		
					Quartile 1	1.00 (Reference)	0.83	
					Quartile 4	1.11 (0.66–1.85)		
Akter [35] 2013 Japan	Cross-sectional 460 subjects Age 21–67 Cases: 59	- Obesity: BMI ≥25 kg/m^2^ - SBP/DBP: ≥130/85 mmHg - FBG: ≥100 mg/dL - TG: ≥150 mg/dL - HDL-c: <40 mg/dL (men); <50 mg/dL (women)	46-item diet history questionnaire PCA Varimax rotation Loading >0.15 3 factors VE 19.5%	***1. Healthy Japanese***: vegetables, fruit, soy products, mushrooms, green tea	Tertile 1 Tertile 3	1.00 (Reference) 1.35 (0.55–3.30)	0.43	Age, sex, workplace, occupational physical activity, job position, marital status, non-occupational physical activity, smoking
				***2. Animal food***: fish and shellfish, meat, processed meat, mayonnaise, and egg	Tertile 1 Tertile 3	1.00 (Reference) 1.54 (0.73–3.24)	0.25	
				***3. Westernized breakfast***: bread, confectioneries, milk and yogurt, mayonnaise, and egg	Tertile 1 Tertile 3	1.00 (Reference) 0.39 (0.16–0.95)	0.02	
Baik [36] 2013 Korea	Cohort 5251 male and female Age 40–69 6-year follow-up Incident cases:1325	- WC: ≥90 cm (men); ≥85 cm (women) - SBP/DBP: ≥130/85 mmHg - FBG: ≥100 mg/dL - TG: ≥150 mg/dL - HDL-c: <40 mg/dL (men); <50 mg/dL (women)	103-item FFQ 27 food groups FA Varimax rotation EIG >2.0 2 factors VE 21%	***1. Healthy***: fish, seafood, vegetables, seaweed, protein foods, fruit, dairy products, and grains	Quintile 1 Quintile 5	1.00 (Reference) 0.76 (0.60–0.97)	<0.05	Age, sex, income, occupation, education, smoking, alcohol intake, quartiles of MET-hours/day, FTO genotypes, and quartiles of energy intake.
				***2. Unhealthy***: refined white rice, meat, sweetened carbonated beverage, and noodles	Quintile 1 Quintile 5	1.00 (Reference) 1.12 (0.92–1.37)	0.38	
Liu [37] 2013 USA	Cross-sectional 1775 African Americans Jackson Heart Study (JHS) Cases: 1053 Age 21–94	- WC: ≥ 90 cm (men); ≥80 cm (women) - SBP/DBP: ≥140/90 mmHg - FBG: ≥100 mg/dL - TG: ≥150 mg/dL - HDL-c: <40 mg/dL (men); <50 mg/dL (women)	158-item FFQ 31 food groups PCA EIG >1.0 Loading > 0.3 3 factors	***1. Southern***: beans and legumes, corn products, fried fish and chicken, meat, processed meat, margarine, butter, rice and pasta	Tertile 1 Tertile 3	1.00 (Reference) 2.16 (1.30–3.60)		Age, sex, smoking and alcohol status, education, and physical activity
				***2. Fast food***: sugar and candy juice, fast food and salty snacks	Tertile 1 Tertile 3	1.00 (Reference) 2.40 (1.40–4.20)		
				***3. Prudent***: fruit and vegetables, cold and hot cereals, nuts and seeds	Tertile 1 Tertile 3	1.00 (Reference) 0.75 (0.50–1.10)		
Naja [38] 2013 Lebanon	Cross-sectional Subjects: 323 Age ≥ 18 Cases:112 Age 42.83 ± 15.34 Controls: 211 Age 36.50 ± 13.91	- WC: ≥94 cm (men); ≥80 cm (women) - SBP/DBP: ≥130/85 mmHg - FBG: ≥100 mg/dL - TG: ≥150 mg/dL - HDL-c: <40 mg/dL (men); <50 mg/dL (women)	61-item FFQ 25 food groups FA Varimax rotation Loading >0.4 3 factors VE 30.62%	***1. Fast Food/Dessert***: fast foods sandwiches, hamburger, shawarma, falafel, pizzas, pies, desserts, carbonated beverages and juices, and mayonnaise	Quintile 1 Quintile 5	1.00 (Reference) 3.13 (1.36–7.22)	0.06	Age, sex, marital status, education, crowding index, physical activity, and smoking
				***2. Traditional Lebanese***: dairy products, olives, fruit, legumes, grains, eggs, vegetable oil, dried fruit, and traditional sweets	Quintile 1 Quintile 5	1.00 (Reference) 1.96 (0.82–4.34)	0.1	
				***3. High-Protein***: fish, chicken, meat, dairy products—low-fat	Quintile 1 Quintile 5	1.00 (Reference) 1.22 (0.54–2.77)	0.76	
Oh [39] 2013 Korea	Cross-sectional KNHANES 5320 subjects Age 30–80 2239 men 3081 women	- WC: ≥90 cm (men); ≥80 cm (women) - SBP/DBP: ≥130/85 mmHg - FBG: ≥110 mg/dL - TG: ≥150 mg/dL - HDL-c: <40 mg/dL (men); <50 mg/dL (women)	24-h dietary recall 33 food groups PCA Varimax rotation EIG >1.5 3 factors	***1. Balanced Korean***: rice, kimchi, whole grains, fish, sea products, vegetables, fruit, dairy products, eggs, meats, and mushrooms.	***MEN***	***MEN***		Age, smoking history, alcohol behavior and physical activity
					Quintile 1	1.00 (Reference)	0.92	
					Quintile 5	0.88 (0.61–1.26)		
					***WOMEN***	***WOMEN***		
					Quintile 1	1.00 (Reference)	<0.05	
					Quintile 5	0.67 (0.47–0.96)		
				***2. Unbalanced Korean***: rice, kimchi and excessive carbohydrate	***MEN***	***MEN***		
					Quintile 1	1.00 (Reference)	0.89	
					Quintile 5	0.99 (0.68–1.45)		
					***WOMEN***	***WOMEN***		
					Quintile 1	1.00 (Reference)	<0.05	
					Quintile 5	1.44 (1.03–2.01)		
				***3. Semi-Western***: meats, poultry, eggs, vegetables, and alcoholic beverages	***MEN***	***MEN***		
					Quintile 1	1.00 (Reference)	0.64	
					Quintile 5	0.95 (0.66–1.39)		
					***WOMEN***	***WOMEN***		
					Quintile 1	1.00 (Reference)	0.17	
					Quintile 5	0.87 (0.63–1.20)		
Hong [40] 2012 Korea	Cross-sectional 406 subjects Age 22–78 Mean 50.6	- WC: ≥90 cm (men); ≥80 cm (women) - SBP/DBP: ≥130/85 mmHg - FBG: ≥110 mg/dL - TG: ≥150 mg/dL - HDL-c: <40 mg/dL (men); <50 mg/dL (women)	24-h recall and a 3-day food record 33 food groups PCA Varimax rotation EIG >1.5 Loading >0.2 4 factors VE 28.8%	***1. Korean traditional***: refined and whole grains, Korean seasonings, onions and garlic, vegetable oil, soy products, starch syrup, and sugar	Quartile 1 Quartile 4	1.00 (Reference) 2.03 (1.05–3.92)	0.047	Age, sex, taking medications, smoking, physical activity, and BMI
				***2. Alcohol and meat***: processed meats, eggs, fish paste, animal fat, and alcohol	Quartile 1 Quartile 4	1.00 (Reference) 1.16 (0.58–2.34)	0.945	
				***3. Sweets and fast foods***: fruit juices, chocolate, ice cream, pizza, and hamburgers	Quartile 1 Quartile 4	1.00 (Reference) 0.81 (0.41–1.61)	0.687	
				***4. Fruit and dairy***: fruit and dairy products, rice cakes and nuts	Quartile 1 Quartile 4	1.00 (Reference) 0.46 (0.22–0.95)	0.025	
Wagner [41] 2012 France	Cross-sectional MONA LISA 3090 subjects Age 35–64 Mean: 50.4 ± 8.4 Cases: 420 men, 259 women Controls: 1141 men, 1271 women	- WC: ≥102 cm (men); ≥88 cm (women) - SBP/DBP: ≥130/85 mmHg - FBG: ≥5.6 mmol/L - TG: ≥1.7 mmol/L - HDL-c: <1.04 mmol/L (men); <1.29 mmol/L (women)	3-day food diary 31 food groups PCA EIG >1.0 Loading >0.2 2 factors VE 13.3%	***1. Energy-dense***: red meat, potatoes, vegetable oils, alcohol, delicatessen products, sodas and sauce	***MEN***	***MEN***		Age, center, educational level, smoking, total calorie intake, time spent sitting, physical activity, heart rate, menopause, BMI
					Quartile 1	1.00 (Reference)	<0.04	
					Quartile 4	1.63 (1.03–2.56)		
					***WOMEN***	***WOMEN***		
					Quartile 1	1.00 (Reference)	0.1	
					Quartile 4	1.53 (0.88–2.66)		
				***2. Convenience-food***: pizza, prepared dishes, cake, cream, grains, junk food, sodas and fruit juices	***MEN***	***MEN***		
					Quartile 1	1.00 (Reference)		
					Quartile 4	0.82 (0.53–1.28)		
					***WOMEN***	***WOMEN***		
					Quartile 1	1.00 (Reference)	0.16	
					Quartile 4	0.69 (0.39–1.24)		
Cho [43] 2011 Korea	Cross-sectional 4984 women Age 30–79	- WC: ≥88 cm - SBP/DBP: ≥130/85 mmHg - FBG: ≥110 mg/dL - TG: ≥150 mg/dL - HDL-c:<50 mg/dL	FFQ 16 food groups FA Varimax rotation Loading >0.2 3 factors VE 35.8%	***1. Western***: fast foods, animal fat-rich foods, fried foods, grilled meat and seafoods, and sweet foods	Quartile 1 Quartile 4	1.00 (Reference) 0.87 (0.54–1.20)	0.304	Age
				***2. Healthy***: green-yellow vegetables, healthy-protein foods, seaweeds, and bonefish	Quartile 1 Quartile 4	1.00 (Reference) 0.58 (0.50–0.91)	0.012	
				***3. Traditional***: salted vegetables and seafoods, cereals, and light-colored vegetables	Quartile 1 Quartile 4	1.00 (Reference) 1.05 (0.79–1.40)	0.873	
Heidemann [44] 2011 Germany	Cross-sectional 4025 subjects Age 18–79	- WC: ≥102 cm (men); ≥88 cm (women) - SBP/DBP: ≥130/85 mmHg - FBG: ≥110 mg/dL - TG: ≥150 mg/dL - HDL-c: <40 mg/dL (men); <50 mg/dL (women)	2678 items 4 weeks face-to-face dietary history 133 food groups PCA Varimax rotation EIG >1.0 2 factors	***1. Processed foods***: refined grains, processed meat, red meat, high-sugar beverages, eggs, potatoes, beer, sweets and cakes, snacks and butter	Quintile 1 Quintile 5	1.00 (Reference) 1.64 (1.10–2.43)	0.001	Age, sex, total energy intake, socioeconomic status, sport activity, smoking
				***2. Health-conscious***: cruciferous vegetables, fruity vegetables, leafy vegetables, all other vegetables, vegetable oils, legumes, fruit, fish and whole grains	Quintile 1 Quintile 5	1.00 (Reference) 0.98 (0.72–1.34)	0.67	
Kim [45] 2011 Korea	Cross-sectional second and third KNHANES 9850 adults Age 19 ≥	- WC: ≥90 cm (men); ≥80 cm (women) - SBP/DBP: ≥130/85 mmHg - FBG: ≥100 mg/dL - TG: ≥150 mg/dL - HDL-c: <40 mg/dL (men); <50 mg/dL (women)	24-h recall 23 food groups FA Varimax rotation EIG >1.0 4 factors VE 26.7%	***1. White rice and kimchi***: White rice, kimchi, vegetables	Tertile 1 Tertile 3	1.00 (Reference) 0.97 (0.85–1.11)	0.61	Age, sex, BMI, energy intake, alcohol intake, smoking status, and physical activity
				***2. Meat and alcohol***: noodles and dumplings, meat and its products, alcohol	Tertile 1 Tertile 3	1.00 (Reference) 1.04 (0.91–1.19)	0.6	
				***3. High-fat, sweets, and coffee***: sugar and sweets, eggs, oils, coffee	Tertile 1 Tertile 3	1.00 (Reference) 1.04 (0.93–1.17)	0.51	
				***4. Grains, vegetables, and fish***: grains, nuts, vegetables, fish and shellfish, seasonings	Tertile 1 Tertile 3	1.00 (Reference) 0.86 (0.76–0.98)	0.02	
Amini [46] 2010 Iran	Cross-sectional 425 subjects Age 35–55	- WC: ≥102 cm (men); ≥88 cm (women) - SBP/DBP: ≥135/85 mmHg - FBG: ≥110 mg/dL - TG: ≥150 mg/dL - HDL-c: <40 mg/dL (men); <50 mg/dL (women)	39-item FFQ PCA Varimax rotation EIG ≥1.5 5 factors VE 26.4%	***1. Western***: sweets, butter, soda, mayonnaise, sugar, cookies, tail of a lamb, hydrogenated fat, eggs	Tertile 1 Tertile 3	1.00 (Reference) 2.32 (1.27–4.21)	0.006	Age, sex, education, and physical activity
				***2. Prudent***: fish, peas, honey, nuts, juice, dry fruit, vegetable oil, liver and organic meat, coconuts	Tertile 1 Tertile 3	1.00 (Reference) 0.58 (0.32–1.04)	0.06	
				***3. Vegetarian***: potatoes, legumes, fruit rich in vitamin C, rice, green leafy vegetables, and fruit rich in vitamin A	Tertile 1 Tertile 3	1.00 (Reference) 1.36 (0.78–2.38)	0.27	
				***4. High-fat dairy***: high-fat yogurt and high-fat milk	Tertile 1 Tertile 3	1.00 (Reference) 1.25 (0.71–2.29)	0.4	
				***5. Chicken and plant***: chicken, fruit rich in vitamin A, green leafy vegetables, mayonnaise	Tertile 1 Tertile 3	1.00 (Reference) 1.05 (0.6–1.84)	0.84	
Denova–Gutierrez [47] 2010 Mexico	Cross-sectional HWCS (Health Workers Cohort Study) 5240 subjects Age 20–70	- WC: ≥102 cm (men); ≥88 cm (women) - SBP/DBP: ≥130/85 mmHg - FBG: ≥100 mg/dL - TG: ≥150 mg/dL - HDL-c: <40 mg/dL (men); <50 mg/dL (women)	116-item FFQ 28 food groups FA Varimax rotation EIG >1.5 Loading ≥0.3 3 factors VE 20.6%	***1. Prudent***: processed vegetable juices, potatoes, fresh fruit, fresh vegetables, legumes	Tertile 1 Tertile 3	1.00 (Reference) 0.99 (0.85–1.17)	0.9	Age, sex, smoking, physical activity, weight change, place of residence, estrogen use, menopausal status, energy intake
				***2. Western***: pastries, refined cereals, corn tortillas, soft drinks	Tertile 1 Tertile 3	1.00 (Reference) 1.58 (1.35–1.85)	0.001	
				***3. High-protein/fat***: red meat, processed meat, margarine (saturated fats), eggs	Tertile 1 Tertile 3	1.00 (Reference) 1.18 (1.01–1.39)	0.04	
DiBello [48] (A) 2009 Samoan Islands	Cross-sectional American Samoan (*n* = 723)	- WC: ≥102 cm (men); ≥88 cm (women) - SBP/DBP: ≥130/85 mmHg - FBG: ≥5.5 mmol/L - TG: ≥1.7 mmol/L - HDL-c: <1.0 mmol/L (men); <1.3 mmol/L (women)	42-item FFQ 13 food groups “Partial least squares regression” 3 factors	***1. Neo-traditional***: crab and lobster, fish, coconut cream dishes, papaya soup, coconut milk, papaya, and taro	Quintile 1 Quintile 5	1.00 (Reference) 0.89 (0.72–1.06)	0.23	Age, sex, modern lifestyle score, smoking, physical activity, total energy intake
				***2. Factor 2***: meat and coconut products such as coconut cream dishes and lamb	Quintile 1 Quintile 5	1.00 (Reference) 0.99 (0.81–1.23)	0.64	
				***3. Modern***: sausage, eggs, milk, cheese, coconut cream, rice, instant noodle soup, bread, pancakes, cereal, butter/margarine, cake, potato chips	Quintile 1 Quintile 5	1.00 (Reference) 1.13 (0.93–1.38)	0.08	
DiBello [48] (B) 2009 Samoan Islands	Cross-sectional Samoan (*n* = 785) Age >18	- WC: ≥102 cm (men); ≥88 cm (women) - SBP/DBP: ≥130/85 mmHg - FBG: ≥5.5 mmol/L - TG: ≥1.7 mmol/L - HDL-c: <1.0 mmol/L (men); <1.3 mmol/L (women)	42-item FFQ 13 food groups “Partial least squares regression” 3 factors	***1. Neo-traditional***: crab and lobster, ripe coconut, coconut cream and coconut cream dishes, and papaya soup	Quintile 1 Quintile 5	1.00 (Reference) 0.74 (0.54–1.01)	0.13	Age, sex, modern lifestyle score, smoking, physical activity, total energy intake
				***2. Factor 3***: meat and coconut products such as coconut cream dishes and lamb	Quintile 1 Quintile 5	1.00 (Reference) 0.98 (0.71–1.35)	0.99	
				***3. Modern***: sausage, eggs, rice, instant noodle soup, pancakes, cereal, papaya, cake, potato chips, ripe coconut, chop suey, rice dishes, crackers, and soup with vegetables	Quintile 1 Quintile 5	1.00 (Reference) 1.21 (0.93–1.57)	0.05	
Noel [49] 2009 USA	Cross-sectional 1167 Puerto Ricans Age 45–75	- WC: ≥102 cm (men); ≥88 cm (women) - SBP/DBP: ≥130/85 mmHg - FBG: ≥5.6 mmol/L - TG: ≥1.7 mmol/L - HDL-c: <1.0 mmol/L (men); <1.3 mmol/L (women)	126-item FFQ 34 food groups PCA Varimax rotation Loading ≥0.2 3 factors	***1. Meat and French fries***: meat, processed meat, French fries, pizza and Mexican foods, eggs, alcohol, and other grains and pasta	Quintile 1 Quintile 5	1.00 (Reference) 1.20 (0.76–2.00)		Age, sex, smoking, alcohol use, education, physical activity, total energy, acculturation, lipid-lowering medication and multivitamin use, BMI
				***2. Traditional***: beans and legumes, rice, oil, vegetables	Quintile 1 Quintile 5	1.00 (Reference) 1.70 (1.04–2.70)		
				***3. Sweets***: candy, sugar and chocolate candy, soft drinks, sugary beverages, sweet baked goods, dairy desserts, and salty snacks	Quintile 1 Quintile 5	1.00 (Reference) 1.30 (0.83–2.10)		
Lutsey [50] 2008 USA	Cohort ARIC 9514 participants Age mean: 53.6 Follow-up 9 3782 incident cases	- WC: ≥102 cm (men); ≥88 cm (women) - SBP/DBP: ≥130/85 mmHg - FBG: ≥100 mg/dL - TG: ≥150 mg/dL - HDL-c: <40 mg/dL (men); <50 mg/dL (women)	66-item FFQ 29 food groups PCA Varimax rotation EIG >2.0 Loading ≥0.2 2 factors VE 19.9%	***1. Western***: refined grains, processed meat, fried foods, and red meat	Quintile 1 Quintile 5	1.00 (Reference) 1.18 (1.03–1.37)	0.03	Age, sex, race, education, center, total calories, smoking and physical activity
				***2. Prudent***: cruciferous and carotenoid vegetables, fruit, fish, and poultry	Quintile 1 Quintile 5	1.00 (Reference) 1.07 (0.95–1.20)	0.11	
Esmaillzadeh [51] 2007 Iran	Cross-sectional 486 Women Age 40–60	- WC: ≥88 cm - SBP/DBP: ≥130/85 mmHg - FBG: ≥110 mg/dL - TG: ≥150 mg/dL - HDL-c: <50 mg/dL	168-item FFQ (IA) 41 food groups PCA Varimax rotation EIG >1.0 Loading ≥0.2 3 factors	***1. Healthy***: fruit, tomatoes, poultry, legumes, cruciferous and green leafy vegetables, other vegetables, tea, fruit juices, and whole grains	Quintile 1 Quintile 5	1.00 (Reference) 0.69 (0.36–0.92)	<0.01	Age, smoking, physical activity, current estrogen use, menopausal status, and family history of diabetes and stroke, energy intake, BMI
				***2. Western***: refined grains, red meat, butter, processed meat, high-fat dairy products, sweets and desserts, pizza, potatoes, eggs, hydrogenated fats, and soft drinks	Quintile 1 Quintile 5	1.00 (Reference) 1.60 (1.06–1.88)	<0.01	
				***3. Traditional***: refined grains, potatoes, tea, whole grains, hydrogenated fats, legumes, and broth	Quintile 1 Quintile 5	1.00 (Reference) 1.07 (0.86–1.22)	0.11	
Panagiotakos [52] 2007 Greece	Cross-sectional ATTICA study 1518 men Age 46 ± 13 1524 women Age 45 ± 13	- WC: ≥102 cm (men); ≥88 cm (women) - SBP/DBP: ≥130/85 mmHg - FBG: ≥100 mg/dL - TG: ≥150 mg/dL - HDL-c: <40 mg/dL (men); <50 mg/dL (women)	156-item FFQ (SA) 22 food groups Varimax rotation PCA EIG >1.0 Loading >0.4 6 factors VE 56%	***1. Healthful***: fish, vegetables, legumes, cereals, and fruit	Logistic regression analysis	0.87 (0.79–0.97)	0.013	Smoking, years of school, income, use of medication, BMI
				***2. High glycemic index and high-fat***: red or white meat and meat products, and potatoes		1.13 (1.05–1.21)	0.004	
				***3. Component***: bread, pasta		0.97 (0.87–1.08)	0.564	
				***4. Component***: dairy, eggs		1.04 (0.93–1.15)	0.516	
				***5. Component***: sweets		1.06 (0.96–1.18)	0.268	
				***6. Component***: alcoholic beverages		1.26 (1.21–1.33)	0.001	

^1^ Waist Circumference (WC); ^2^ Systolic Blood Pressure (SBP)/Diastolic Blood Pressure (DBP); ^3^ Fasting Blood Glucose (FBG); ^4^ Triglyceride (TG); ^5^ HDL cholesterol (HDL-c); ^6^ Food Frequency Questionnaire (FFQ); ^7^ Interviewer Administered (IA); ^8^ Factor Analysis (FA); ^9^ Eigenvalues (EIG); ^10^ Variance Explained (VE); ^11^ Body Mass Index (BMI); ^12^ Principal Component Analysis (PCA); ^13^ Not Reported (NR); ^14^ Reduced Rank Regression (RRR); ^15^ Cluster Analysis (CA); ^16^ Principal Component Factor Analysis (PCFA); ^17^ Self-Administered (SA).

**Table 2:** Results of stratified analysis of the Metabolic Syndrome risk estimates for the highest compared with the lowest intake categories of “Healthy” and “Meat/Western” dietary patterns ^a,b^.

	Combined Risk Estimate		Test of Heterogeneity			Publication Bias	
Dietary Patterns	Value (95% CI)	*p*	Q	I^2^%	*p*	P (Egger Test)	P (Begg Test)
**“Healthy”**							
All (*n* = 42) ^c^	0.85 (0.79–0.91)	<0.0001	132.11	68.97	<0.0001	0.005	0.074
Excluding: Bell [27], Arisawa [33] and Panagiotakos [52] (*n* = 39) ^d^	0.84 (0.77–0.91)	<0.0001	110.23	65.53	<0.0001	0.011	0.088
Study design							
Cohort studies (*n* = 3)	0.76 (0.50–1.15)	0.195	21.58	90.73	<0.0001	0.081	0.117
Cross-sectional studies (*n* = 39)	0.86 (0.79–0.92)	<0.0001	110.22	65.52	<0.0001	0.016	0.097
Geographic location							
Eastern countries (*n* = 28)	0.78 (0.71–0.86)	<0.0001	63.57	57.53	<0.0001	0.098	0.343
Western countries (*n* = 14)	0.97 (0.88–1.07)	0.557	39.61	67.18	0.0002	0.255	0.208
Geographic area							
Asia (*n* = 27)	0.77 (0.70–0.85)	<0.0001	57.22	54.56	0.0003	0.215	0.466
Europe (*n* = 6)	0.92 (0.81–1.04)	0.188	10.70	53.27	0.058	0.952	0.851
America (*n* = 7)	0.98 (0.84–1.15)	0.806	15.43	61.12	0.017	0.272	0.099
Sex							
Women (*n* = 8)	0.74 (0.59–0.92)	0.007	22.95	69.50	0.002	0.422	0.322
Men (*n* = 5)	0.85 (0.73–0.99)	0.032	2.81	0.00	0.589	0.831	1.000
**“Meat/Western”**							
All (*n* = 40)	1.19 (1.09–1.29)	<0.0001	158.62	75.41	<0.0001	0.121	0.155
Excluding: Bell [27], Arisawa [33] and Panagiotakos [52] (*n* = 37) ^d^	1.21 (1.10–1.34)	<0.0001	146.92	75.50	<0.0001	0.151	0.209
Study design							
Cohort studies (*n* = 4)	1.24 (1.08–1.41)	0.002	4.99	39.84	0.173	0.911	1.000
Cross-sectional studies (*n* = 36)	1.18 (1.08–1.30)	0.0004	149.62	76.61	<0.0001	0.119	0.120
Geographic location							
Eastern countries (*n* = 26)	1.17 (1.05–1.32)	0.006	77.75	67.85	<0.0001	0.021	0.193
Western countries (*n* = 14)	1.21 (1.06–1.38)	0.004	77.65	83.26	<0.0001	0.471	0.477
Geographic area							
Asia (*n* = 25)	1.20 (1.08–1.33)	0.001	53.66	55.28	0.0005	0.100	0.112
Europe (*n* = 7)	1.15 (1.03–1.31)	0.014	14.88	59.68	0.021	0.682	0.881
America (*n* = 6)	1.33 (1.00–1.77)	0.047	39.30	87.28	<0.0001	0.970	0.348
Gender							
Women (*n* = 7)	1.01 (0.82–1.23)	0.945	13.47	55.47	0.036	0.481	0.293
Men (*n* = 4)	1.21 (0.89–1.65)	0.226	6.96	56.91	0.073	0.163	0.042

^a^ The analysis was performed when several data ≥3 were available; ^b^ The risk estimates were calculated using the random-effect model; ^c^ In brackets are indicated the number of data included in the analysis; ^d^ Studies were the risk was calculated on the base of one standard deviation increment.
